# An efficient exact algorithm for computing all pairwise distances between reconciliations in the duplication-transfer-loss model

**DOI:** 10.1186/s12859-019-3203-9

**Published:** 2019-12-17

**Authors:** Santi Santichaivekin, Ross Mawhorter, Ran Libeskind-Hadas

**Affiliations:** Department of Computer Science, Harvey Mudd College, Claremont, CA US

**Keywords:** Phylogenetic trees, Maximum parsimony reconciliation, Duplication-transfer-loss model

## Abstract

**Background:**

Maximum parsimony reconciliation in the duplication-transfer-loss model is widely used in studying the evolutionary histories of genes and species and in studying coevolution of parasites and their hosts and pairs of symbionts. While efficient algorithms are known for finding maximum parsimony reconciliations, the number of reconciliations can grow exponentially in the size of the trees. An understanding of the space of maximum parsimony reconciliations is necessary to determine whether a single reconciliation can adequately represent the space or whether multiple representative reconciliations are needed.

**Results:**

We show that for any instance of the reconciliation problem, the distribution of pairwise distances can be computed exactly by an efficient polynomial-time algorithm with respect to several different distance metrics. We describe the algorithm, analyze its asymptotic worst-case running time, and demonstrate its utility and viability on a large biological dataset.

**Conclusions:**

This result provides new insights into the structure of the space of maximum parsimony reconciliations. These insights are likely to be useful in the wide range of applications that employ reconciliation methods.

## Background

Phylogenetic tree reconciliation is an important method for studying the evolutionary histories of pairs of taxa such as genes and species, parasites and their hosts, and pairs of symbiont species. Given two phylogenetic trees and the association of their extant taxa, the objective is to find an association of the two trees that best explains their incongruity using a biological model of evolutionary events. In the widely-used Duplication-Transfer-Loss (DTL) model, the events considered are contemporaneous speciation, duplication, host/horizontal gene transfer, and loss/extinction.

Reconciliation is typically performed in a maximum parsimony framework in which each type of event is assigned a non-negative cost and the objective is to find a mapping of one tree (e.g., the gene tree or parasite tree) onto the other tree (e.g., the species tree or host tree) that minimizes the total sum of the costs of the constituent events. A reconciliation of minimum cost is called a *maximum parsimony reconciliation (MPR)*. Although reconciliation is also possible in probabilistic frameworks, the underlying algorithms are generally prohibitively slow and are particularly sensitive to the choice of their many parameters.

While a single maximum parsimony reconciliation can be found in polynomial time [1–3], the number of MPRs can grow exponentially with the size of the trees [1, 4]. For example, in a benchmark Tree of Life dataset with 100 primarily prokaryotic species [5], over 10% of gene families had more than 10^5^ MPRs and some gene families induced over 10^100^ MPRs [1]. Moreover, the choice of event costs can have a significant impact on the space of MPRs [6]. Consequently, making inferences from a single MPR may lead to conclusions that are not supported, or even contradicted, by other MPRs.

It is important, therefore, to understand the structure and diversity of MPR space. For example, if MPRs are largely similar to one another, then a single MPR may suffice to make robustly supported conclusions. In other cases, MPR space may be so diverse that conclusions drawn from a single MPR, or even a sample of MPRs, may not be reliable.

A number of prior studies have explored the size and structure of MPR space. The number of MPRs can be computed in polynomial time as a byproduct of computing a maximum parsimony reconciliation [1–3]. Using a compact representation of MPR space called the *reconciliation graph* [7], Nguyen et al. [8] showed how a single “median” MPR can be computed in polynomial time. Ozdemir et al. [9] showed that this result can be generalized to find a set of *k* medoids or *k* centers that represent MPR space. Ma et al. [10] gave a polynomial-time 2-approximation algorithm for covering MPR space with a set of MPRs that, collectively, contain all of the events that arise in MPR space. Haack et al. [11] showed how the diameter of MPR space can be computed in polynomial time and demonstrated that, in many cases, MPR space is very diverse.

Recently, Huber et al. [12] proposed computing the distribution of pairwise distances between MPRs, with respect to a given distance metric, as a tool for obtaining a deeper understanding of MPR space than was previously possible. If, for example, the pairwise distances between MPRs tend to be small, then choosing a single MPR to represent that space may be justifiable. However, if the pairwise distances are large or the distribution of the distances is multimodal, then the conclusions drawn from a single MPR are likely to be less robust.

Since MPR space is large, the approach proposed by Huber et al. selects a sample of MPRs and iteratively computes their pairwise distances. Because the number of pairs grows quadratically with the sample size, this approach is only viable for small sample sizes, providing a potentially coarse approximation of the true distribution. The problem of whether the exact distribution of pairwise distances across the entire space of MPRs can be computed efficiently was left open [12].

We solve this problem by showing how the pairwise distances between all pairs of MPRs can be computed exactly, without sampling, in time polynomial in the size of the trees. For concreteness, our presentation uses the symmetric distance metric [8] which measures the distance between two reconciliations as the number of events that are found in one reconciliation or the other, but not both. We show that our results are easily extendible to other distance metrics as well. Importantly, the asymptotic running time is not a function of the number of MPRs. In practice, our algorithm computes this distribution in seconds, even for problem instances inducing over 10^100^ MPRs.

Using a Tree of Life dataset [5], we show that the distributions of pairwise distances can vary dramatically across problem instances and are also sensitive to the event costs. We believe that the *PairTree* software that accompanies this paper will provide an important tool when performing analyses using DTL maximum parsimony reconciliation.

In summary, the contributions of this paper are:

1. An efficient polynomial-time algorithm for computing the pairwise distances between all MPRs for any instance of the DTL reconciliation problem;
2. Experimental results that demonstrate both the speed of the algorithm on real datasets and its utility in obtaining new insights into the space of MPRs; and
3. A Python implementation of our algorithm in the *PairTree* package (www.cs.hmc.edu/~hadas/pairtree).

### Maximum parsimony reconciliations

A DTL-MPR instance is a 6-tuple (*S*,*G*,*ϕ*,*d*,*t*,*ℓ*) where *S*=(*V*_*S*_,*E*_*S*_) and *G*=(*V*_*G*_,*E*_*G*_) are binary trees, *ϕ* is a mapping from the leaves of *G* to the leaves of *S* (the mapping need not be one-to-one nor onto), and *d*, *t*, and *ℓ* are non-negative costs corresponding to duplication, transfer, and loss events, respectively, which are explained below. We assume that the trees are undated, but all results in this paper can be easily adapted to dated trees as well.

A *reconciliation mapping* for a given instance is a mapping *Φ* from the vertices of *G* to the vertices of *S* such that *Φ*(*g*)=*ϕ*(*g*) for each leaf *g* of *G* and, if *g* is an internal vertex of *G* with children *g*^′^ and *g*^′′^, then (1) *Φ*(*g*) cannot be a descendant of either *Φ*(*g*^′^) or *Φ*(*g*^′′^) and (2) at least one of *Φ*(*g*^′^) or *Φ*(*g*^′′^) is equal to or a descendant of *Φ*(*g*).

A reconciliation mapping induces four types of events: Each internal vertex *g*∈*V*_*G*_ induces exactly one speciation, duplication, or transfer event and zero or more loss events. For an internal gene tree vertex *g*, with children *g*^′^ and *g*^′′^, the events induced by *Φ* are as follows:

- **Speciation event:** Vertex *g* induces a speciation event if one of *Φ*(*g*^′^) and *Φ*(*g*^′′^) is in the left subtree and the other is in the right subtree of *Φ*(*g*).
- **Duplication event:** Vertex *g* induces a duplication event if each of *Φ*(*g*^′^) and *Φ*(*g*^′′^) is either equal to or a descendant of *Φ*(*g*) but does not satisfy the requirements for a speciation event.
- **Transfer event:** Vertex *g* induces a transfer event if exactly one of *Φ*(*g*^′^) and *Φ*(*g*^′′^) is either equal to or a descendant of *Φ*(*g*) and the other is neither an ancestor nor a descendant of *Φ*(*g*).
- **Loss events:** Each non-root vertex *g* (including leaf vertices) may induce zero or more loss events as follows: Let *p*(*g*) denote the parent of *g* in tree *G*. If *Φ*(*p*(*g*)) is ancestral to *Φ*(*g*), then each species vertex *s* on the path from *Φ*(*p*(*g*)) to *Φ*(*g*) induces a loss event, except for *Φ*(*g*) and also not *Φ*(*p*(*g*)) if *p*(*g*) induces a speciation event. For each loss induced by a vertex *s* on the path from *Φ*(*p*(*g*)) to *Φ*(*g*), we say that *gpasses* through *s*.

**Fig. 1:**
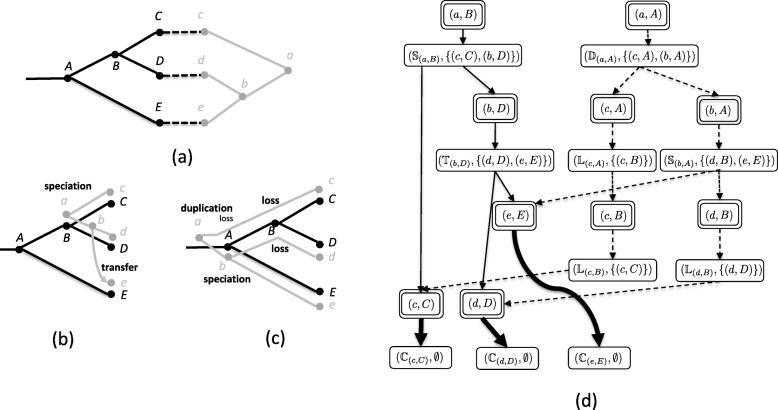
DTL reconciliation. **a** An instance of the DTL reconciliation problem comprising a species tree (black), a gene tree (gray), and a leaf mapping. Duplication, transfer and loss costs are 1, 4, and 1, respectively. **b** and **c** Two different MPRs, each with total cost 4. **d** The associated reconciliation graph. Mapping nodes are indicated with double line borders. Event nodes are designated with $\mathbb {S}$ (speciation event), $\mathbb {D}$ (duplication event), $\mathbb {T}$ (transfer event), or $\mathbb {L}$ (loss event). The reconciliation traversal indicated by solid edges corresponds to the MPR in (**b**) and the reconciliation traversal indicated by dashed edges corresponds to the MPR in (**c**); bold edges indicate shared elements of the two MPRs. Figure adapted from Haack et. al [11] with permission

The cost of a reconciliation mapping is the sum of the number of events of each type, weighted by their event costs. Speciations are generally considered null events and thus have cost zero. A reconciliation mapping of minimum cost is called a *maximum parsimony reconciliation (MPR)*. Figure 1a shows an example of a DTL-MPR instance and Fig. 1b, c shows two different MPRs for that instance using duplication, transfer, and loss costs of 1, 4, and 1, respectively.

An MPR can be found in time *O*(|*G*||*S*|) [1, 13], although the problem becomes NP-complete if the reconciliation is required to be temporally feasible in the sense that there exists a total ordering of the events such that an event involving a gene vertex *g* comes earlier in the ordering than any event involving a descendant of *g*. Fortunately, temporal infeasiblity can be detected when it occurs [13, 14] and experimental results suggest that it is not common [14, 15].

### Reconciliation graphs and traversals

The space of all MPRs can be compactly represented using a *reconciliation graph* (Fig. 1d). This representation was originally developed by Scornavacca et al. [7] for dated trees and later modified and adapted for undated trees [10]. For completeness, this representation is summarized below.

Consider a DTL-MPR instance (*S*,*G*,*ϕ*,*d*,*t*,*ℓ*). Let ***Φ*** denote the set of all MPRs for this instance. For a gene vertex *g*, let the children of *g* be denoted by *g*^′^ and *g*^′′^. Then, **events**(*g*,*s*) is the set of the following tuples induced by each MPR *Φ*∈***Φ***:

- $(\mathbb {S}_{(g,s)}, \{(g^{\prime }, s^{\prime }), (g^{\prime \prime }, s^{\prime \prime })\})$ for each speciation in which *g* is mapped to *s*, *g*^′^ is mapped to *s*^′^ or one of its descendants, and *g*^′′^ is mapped to *s*^′′^ or one of its descendants, where *s*^′^ and *s*^′′^ denote the children of *s*;
- $(\mathbb {D}_{(g,s)}, \{(g^{\prime }, s), (g^{\prime \prime }, s)\})$ for each duplication in which *g* is mapped to *s*.
- $(\mathbb {T}_{(g,s)}, \{(g^{\prime }, s), (g^{\prime \prime }, \hat {s})\})$ for each transfer in which *g* is mapped to *s* and one child, wlog *g*^′′^, is mapped to a vertex $\hat {s}$ that is not ancestrally related to *s*;
- $(\mathbb {L}_{(g,s)}, \{(g, s^{\prime })\})$ for each loss in which *g* passes through *s*, and *s*^′^ is the vertex that follows *s* on the path from *Φ*(*p*(*g*)) to *Φ*(*g*); and
- $(\mathbb {C}_{(g,s)}, \varnothing)$ for a contemporaneous leaf association where *g* and *s* are leaves and *ϕ*(*g*)=*s*.

We make several observations about this tuple representation of events. First, if *g* is mapped to *s* as a speciation event, the children of *g*, denoted *g*^′^ and *g*^′′^, are mapped to descendents of *s*. However, the speciation event is represented by associating *g*^′^ with one child of *s* (denoted *s*^′^) and associating *g*^′′^ with the other child of *s* (denoted *s*^′′^). Loss events are introduced for each loss incurred as *g*^′^ (or *g*^′′^) passes through species vertices on the path from *s*^′^ (or *s*^′′^) to *Φ*(*g*^′^) (or *Φ*(*g*^′′^)). Similarly, for a duplication event in which *g* is mapped to *s*, the children of *g* may be mapped to *s* or descendants of *s*. However, the duplication event is represented by associating both *g*^′^ and *g*^′′^ with *s* and then loss events are introduced for each loss on the path from *s* to *Φ*(*g*^′^) and on the path from *s* to *Φ*(*g*^′′^). Finally, if *g* is mapped to *s* as a transfer event, then one child of *g*, wlog *g*^′^, is mapped to *g* or one of its descendants while the other child, *g*^′′^ is mapped to a vertex $\hat {s}$ that is not ancestrally related to *s*. The transfer event is represented by associating *g*^′^ with *s* (and associating *g*^′′^ with $\hat {s}$); loss events are introduced for each loss on the path from *s* to *Φ*(*g*^′^).

For each such tuple *e*, let **type**(*e*) denote its first element, namely the event type and the ordered pair (*g*,*s*), and let **associations**(*e*) denote its second element, namely a set of zero or more ordered pairs. Note that if *e* corresponds to a speciation, duplication, or transfer event, then **associations**(*e*) is a set containing two ordered pairs, each representing an association between a gene tree vertex and a species tree vertex. If *e* is a loss event, then **associations**(*e*) is a set containing one such ordered pair indicating where the loss is incurred.

#### Reconciliation graph

The reconciliation graph contains a *mapping node* for each (*g*,*s*) pair where *g* is mapped to *s* in some MPR and, if not already included, a node (*g*,*s*) is also introduced if *g* passes through *s* due to a loss event. The reconciliation graph also contains an *event node* corresponding to each tuple in **events**(*g*,*s*). There is a directed edge from each mapping node (*g*,*s*) to each event node in **events**(*g*,*s*) and a directed edge from each event node *e* to a mapping node corresponding to an ordered pair in **associations**(*e*).(Throughout this paper, we use the term *vertex* for an element of the gene or species tree and the term *node* for an element of the reconciliation graph.)

The representation is compact by merit of the fact that, while a mapping (*g*,*s*) and its events may arise in many different MPRs, they are shared in this graph representation. Therefore, the size of the reconciliation graph is easily seen to be polynomial in the size of the two trees.

Ma et al. give a formal description of the algorithm for constructing undated reconciliation graphs, a derivation of its *O*(|*G*||*S*|^2^) running, and show that undated reconciliation graphs are acyclic [10]. Figure 1d shows the reconciliation graph for the DTL-MPR instance in Fig. 1a when duplication and loss have cost one and transfer has cost four.

#### Reconciliation traversal

Next, we define reconciliation traversals, which correspond to MPRs. Let $\mathbf {sources}(\mathcal {R})$ denote the set of source nodes of reconcilation graph $\mathcal {R}$ which, by definition, are mapping nodes of the form (*rg*,·) where *rg* represents the root of tree *G*.

For a reconciliation graph $\mathcal {R}$, a *reconciliation traversal* (abbreviated as *traversal*) is a subgraph of $\mathcal {R}$ whose root is a mapping node in $\mathbf {sources}(\mathcal {R})$. Each non-leaf mapping node added to the traversal has exactly one of its event node children added to the traversal. Each event node added to the traversal has all of its mapping node children added to the traversal. Figure 1d shows two traversals corresponding to the two MPRs in Fig. 1b, c.

There is a straightforward bijection between the set of MPRs and the set of traversals in the reconciliation graph [10]. A traversal, in turn, can be represented as the set of event nodes that it comprises. Thus, we may represent an MPR as the set of event nodes in the corresponding traversal. For an MPR *R*, let *E*(*R*) denote the set of event nodes in that reconciliation.

The PDV Algorithm described in the next section computes the pairwise distances using a dynamic programming formulation that operates on increasingly larger subgraphs of the reconciliation graph. For any mapping node (*g*,*s*) in the reconciliation graph, the subgraph of all nodes reachable from (*g*,*s*) is called the *reconciliation subgraph* rooted at (*g*,*s*).

The definition of a traversal is also generalized to begin at any mapping node (*g*,*s*) in the reconciliation graph. This is called a *reconciliation subtraversal* rooted at (*g*,*s*). Therefore, a traversal is a subtraversal whose root is in $\mathbf {sources}(\mathcal {R})$.

### Distance

Let *X*⊕*Y* denote the symmetric set difference of sets *X* and *Y*, namely the set of elements that are found in *X* or *Y* but not both. Given two reconciliations *R*_1_ and *R*_2_, the *symmetric set distance* between them, denoted *d*(*R*_1_,*R*_2_) is defined to be |*E*(*R*_1_)⊕*E*(*R*_2_)|. It is easily verified that the symmetric set distance satisfies the requirements of a distance metric.

This distance metric has been used to measure the distance between reconciliations in prior work [7–9, 11, 16] and has been shown to have a number of desirable properties [8, 16]. Other distance metrics are also possible [12, 16, 17] and in a later section we show how our algorithm can be applied to several of those metrics. Henceforth, unless specified otherwise, we use the term “distance” to mean symmetric set distance.

### Vector operations

Throughout this work, vectors are understood to be over the non-negative integers. For a vector *v*, let *v*[*i*] denote the element at index *i*. We use several standard operations on vectors. Given two vectors *u* and *v*, the *sum* of *u* and *v*, denoted *u*+*v* is defined by (*u*+*v*)[*i*]=*u*[*i*]+*v*[*i*]. The *convolution* of *u* and *v*, denoted *u*∗*v* is defined by:

$$(u \ast v) [i] = \sum_{j = 0}^{i} u[j] v[i-j] $$

Given a vector *v*, *σ*^*j*^, the *j*-place *shift* of *v* is defined by:

$$\sigma^{j}(v)[i] = \left\{ \begin{array}{lr} v[i-j] & : i \geq j \\ 0 & : i < j \end{array} \right. $$

Henceforth, we use the shorthand *σ* for *σ*^1^. Note also that *σ*^0^(*v*)=*v*. Finally, for a vector *v* and positive integer *c*, the *scale* of *v* by *c* is defined by (*cv*)[*i*]=*c*×*v*[*i*].

## Computing pairwise distances

For a given DTL-MPR instance *I*=(*S*,*G*,*ϕ*,*d*,*t*,*ℓ*), let ${\mathcal {S}}(I)$ denote the set of all MPRs. The *pairwise distance vector* is the vector *v* such that *v*[*i*] denotes the number of pairs of MPRs in ${\mathcal {S}}(I)$ whose distance is exactly *i*. Note that *v*[0] is exactly the number of MPRs in ${\mathcal {S}}(I)$ since two MPRs *R*_1_ and *R*_2_ are at distance 0 if and only if *R*_1_=*R*_2_. Moreover, the maximum value of *i* such that *v*[*i*]>0 is the maximum distance between any two MPRs, namely the diameter of the space. For convenience in describing the algorithm, we treat these vectors as having infinite dimension. We later establish a bound on the diameter of the space which allows the implementation to use vectors of finite dimension.

### The Algorithm

To compute the pairwise distance vector, we first compute the reconciliation graph as described in the previous section. Consider a DTL-MPR instance *I*=(*S*,*G*,*ϕ*,*d*,*t*,*ℓ*) and its reconciliation graph $\mathcal {R}$. Let **PDV**_*g*_(*s*_1_,*s*_2_) denote the vector *v* such that *v*[*i*] counts the number of pairs of subtraversals, one rooted at (*g*,*s*_1_) and the other rooted at (*g*,*s*_2_), that differ in exactly *i* event nodes. Note that *s*_1_ and *s*_2_ need not be distinct. Since some (*g*,*s*) pairs may not be present as mapping nodes in $\mathcal {R}$, we define **PDV**_*g*_(*s*_1_,*s*_2_) to be the zero vector if either (*g*,*s*_1_) or $(g, s_{2}) \not \in \mathcal {R}$.

Recall that *rg* denotes the root of *G* and each element of $\mathbf {sources}(\mathcal {R})$ is a mapping node of the form (*rg*,·). Since every traversal, and thus every MPR, must begin at an element of $\mathbf {sources}(\mathcal {R})$, the pairwise distance vector for the entire MPR space is:

$$v = \ \underset{(rg, s_{1}), (rg, s_{2}) \in \mathbf{sources}(\mathcal{R})}{\sum} \mathbf{PDV}_{rg}(s_{1}, s_{2}) $$

where the summation represents the vector sum.

We describe a recursive formulation for **PDV**_*g*_(*s*_1_,*s*_2_) which allows us to compute these values efficiently via dynamic programming (DP). In fact, our approach uses mutual recursion which results in multiple DP tables. In addition to the **PDV** table, the two “helper” tables are called **bothSDT** and **topSDT**. We begin by describing the **bothSDT** table.

#### The **bothSDT** table

A speciation, duplication, or transfer event is called an *SDT event* to distinguish it from a loss event. We define **bothSDT**_*g*_(*s*_1_,*s*_2_) as the vector of pairwise distances for the subtraversals rooted at mapping nodes (*g*,*s*_1_) and (*g*,*s*_2_) assuming that the first event nodes in *both* subtraversals are not loss events.

For the base case, if *g* is a leaf of the gene tree, and *s*_1_=*s*_2_, then let *s*=*s*_1_=*s*_2_. If *ϕ*(*g*)=*s*, then:

$$\begin{array}{@{}rcl@{}} \mathbf{bothSDT}_{g}(s, s)[0] &=& 1 \\ \mathbf{bothSDT}_{g}(s, s)[i] &=& 0 : i \geq 1 \end{array} $$

This represents the fact that there is a single subtraversal rooted at (*g*,*s*), namely the subtraversal that maps *g* to *s* and its distance to itself is 0. If *ϕ*(*g*)≠*s* or *s*_1_≠*s*_2_ then **bothSDT**_*g*_(*s*_1_,*s*_2_) is the zero vector since there are no valid pairs of subtraversals rooted at (*g*,*s*_1_) and (*g*,*s*_2_) in those cases.

If *g* is not a leaf, then let the children of *g* be denoted *g*^′^,*g*^′′^ and let **SDTevents**(*g*,*s*) denote the set of speciation, duplication, or transfer (not loss) event node children of the mapping node (*g*,*s*). If *e*∈**SDTevents**(*g*,*s*), the children of *e* will be mapping nodes of the form (*g*^′^,*κ*) and (*g*^′′^,*τ*). Let $\mathbf {spec}_{{g^{\prime }}}({e}) = \kappa $ and $\mathbf {spec}_{{g^{\prime \prime }}}({e}) = \tau $.

Our objective is to count the number of pairs of subtraversals rooted at (*g*,*s*_1_) and (*g*,*s*_2_) that begin with SDT events. (Recall that *s*_1_ and *s*_2_ may be equal.) Let *e*_1_ and *e*_2_ denote such a pair of SDT events. Note that if *e*_1_≠*e*_2_, then these events contribute two to the distance between the subtraversals. If *e*_1_=*e*_2_ (which can only occur if *s*_1_=*s*_2_), then these events do not contribute to the pairwise distance.

Consider a given pair of subtraversals (*A*^′^,*A*^′′^) rooted at $(g^{\prime }, \mathbf {spec}_{{g^{\prime }}}({e_{1}}))$ and $(g^{\prime \prime }, \mathbf {spec}_{{g^{\prime \prime }}}({e_{1}}))$, respectively, and another pair (*B*^′^,*B*^′′^) rooted at $(g^{\prime }, \mathbf {spec}_{{g^{\prime }}}({e_{2}}))$ and $(g^{\prime \prime }, \mathbf {spec}_{{g^{\prime \prime }}}({e_{2}}))$, respectively. In the treatment below, we begin by assuming that *A*^′^≠*B*^′^ and *A*^′′^≠*B*^′′^ and later correct for the cases when one or both pairs may be equal.

To construct a pair of subtraversals rooted at (*g*,*s*_1_) and (*g*,*s*_2_) that result from the events *e*_1_ and *e*_2_ and the subtraversals *A*^′^,*A*^′′^,*B*^′^,*B*^′′^, we first choose a subtraversal for each of event *e*_1_’s two children. Note that *A*^′^ and *A*^′′^ can always be chosen, but if, for example, $\mathbf {spec}_{{g^{\prime }}}({e_{1}}) = \mathbf {spec}_{{g^{\prime }}}({e_{2}})$, then we can also choose *e*_1_ with *B*^′^ and *A*^′′^. In any case, the second subtraversal, which involves *e*_2_, is uniquely induced by the first subtraversal. If *e*_1_ with *A*^′^ and *A*^′′^ are chosen for the first subtraversal then *e*_2_ with *B*^′^ and *B*^′′^ comprise the second subtraversal and if *e*_1_ with *B*^′^ and *A*^′′^ are chosen for the first subtraversal then *e*_2_ with *A*^′^ and *B*^′′^ comprise the second subtraversal.

Since the relationships of *e*_1_ and *e*_2_ and their children dictate what choices of subtraversals are possible, we now enumerate the possible cases. In each case, we define the value of a function **choices**(*e*_1_,*e*_2_) which is used later.

##### Case 1: *e*_1_≠*e*_2_ and their four mapping node children are all distinct.

In this case, exactly one pair of subtraversals is induced: *e*_1_,*A*^′^,*A*^′′^ with *e*_2_,*B*^′^,*B*^′′^. Thus, **choices**(*e*_1_,*e*_2_)=1.

##### Case 2: *e*_1_≠*e*_2_ and exactly one mapping node child is shared.

In this case, two pairs of subtraversals are induced. Without loss of generality, assume that $\mathbf {spec}_{{g^{\prime }}}({e_{1}}) = \mathbf {spec}_{{g^{\prime }}}({e_{2}})$. Then, the two pairs of subtraversals are *e*_1_,*A*^′^,*A*^′′^ with *e*_2_,*B*^′^,*B*^′′^ and *e*_1_,*B*^′^,*A*^′′^ with *e*_2_,*A*^′^,*B*^′′^. Thus, **choices**(*e*_1_,*e*_2_)=2.

##### Case 3: *e*_1_≠*e*_2_ and both pairs of mapping node children are shared.

In this case, four pairs of subtraversals are induced: *e*_1_,*A*^′^,*A*^′′^ with *e*_2_,*B*^′^,*B*^′′^, *e*_1_,*A*^′^,*B*^′′^ with *e*_2_,*B*^′^,*A*^′′^, *e*_1_,*B*^′^,*A*^′′^ with *e*_2_,*A*^′^,*B*^′′^, and *e*_1_,*B*^′^,*B*^′′^ with *e*_2_,*A*^′^,*A*^′′^. Thus, **choices**(*e*_1_,*e*_2_)=4.

##### Case 4: *e*_1_=*e*_2_.

In this case, two pairs of subtraversals are induced. Letting *e*=*e*_1_=*e*_2_, they are *e*,*A*^′^,*A*^′′^ with *e*,*B*^′^,*B*^′′^ and *e*,*A*^′^,*B*^′′^ with *e*,*B*^′^,*A*^′′^. Thus, **choices**(*e*_1_,*e*_2_)=2.

Recall that the *i*^*th*^ element of vector **bothSDT**_*g*_(*s*_1_,*s*_2_) denotes the number of pairs of subtraversals, one rooted at (*g*,*s*_1_) and the other rooted at (*g*,*s*_2_), that begin with SDT events and whose distance is exactly *i*. Let $u = \mathbf {PDV}_{g^{\prime }}(\mathbf {spec}_{{g^{\prime }}}({e_{1}}), \mathbf {spec}_{{g^{\prime }}}({e_{2}}))$ and $v = \mathbf {PDV}_{g^{\prime \prime }}(\mathbf {spec}_{{g^{\prime \prime }}}({e_{1}}), \mathbf {spec}_{{g^{\prime \prime }}}({e_{2}}))$. Observe that $\sum _{k=0}^{j} \mathbf {choices}(e_{1}, e_{2}) \times u[k] \times v[j-k]$ counts the number of pairs of subtraversals rooted at (*g*,*s*_1_) and (*g*,*s*_2_), using *e*_1_ and *e*_2_, that differ in exactly *j* events, without the distance contribution of *e*_1_,*e*_2_ if *e*_1_≠*e*_2_.

The above case analysis, and thus the value of **choices**(*e*_1_,*e*_2_), assumes that *A*^′^≠*B*^′^ and *A*^′′^≠*B*^′′^. If one of *A*^′^=*B*^′^ or *A*^′′^=*B*^′′^ then we have overcounted by a factor of two and must adjust accordingly. If both *A*^′^=*B*^′^ and *A*^′′^=*B*^′′^, we must divide the count by a factor of four. Note that *A*^′^=*B*^′^ and *A*^′′^=*B*^′′^ correspond to a pair of subtraversals counted in *u*[0] and *v*[0], respectively.

These observations allow us to compute **bothSDT**_*g*_(*s*_1_,*s*_2_) using vector convolution, addition, and scaling. First, for a given vector *u*, define *u*_0_ to be the vector that is identical to *u* at index 0 and is zero at all other indices. Let *u*_−0_ denote the vector that is zero at index 0 and is identical to *u* at all other indices. Letting *c*=**choices**(*e*_1_,*e*_2_), define the *scaled convolution* operator ∗_*c*_ as:

1 $$ {}u \ast_{c} v = c (u_{-0} \ast v_{-0}) + \left\lceil \frac{c}{2} \right\rceil (u_{0} \ast v_{-0} + u_{-0} \ast v_{0}) + \left\lceil \frac{c}{4} \right\rceil (u_{0} \ast v_{0})  $$

Note that the term *c*(*u*_−0_∗*v*_−0_) accounts for the case that *A*^′^≠*B*^′^ and *A*^′′^≠*B*^′′^, the next term accounts for the case that exactly one of *A*^′^=*B*^′^ or *A*^′′^=*B*^′′^, and the last term accounts for the case that *A*^′^=*A*^′′^ and *B*^′^=*B*^′′^. The ceilings $\left \lceil \frac {c}{2} \right \rceil $ and $\left \lceil \frac {c}{4} \right \rceil $ are used so that the corresponding distance vectors are not omitted when *c* is either 1 or 2.

For two events *e*_1_,*e*_2_ we define *δ*(*e*_1_,*e*_2_)=1 if *e*_1_≠*e*_2_ and 0 otherwise, we can now compute **bothSDT**_*g*_(*s*_1_,*s*_2_) by summing Eq. 1 over all event pairs *e*_1_,*e*_2_, and shifting the distance vector by 2 if *e*_1_≠*e*_2_ to account for the difference in those two events:

$$\begin{array}{@{}rcl@{}} \mathbf{bothSDT}_{g}(s_{1}, s_{2}) & = &  \\ & & {\underset{\substack{e_{1} \in \mathbf{SDTevents}(g,s_{1})\\ e_{2} \in \mathbf{SDTevents}(g, s_{2})\\k=\delta(e_{1},e_{2})}}{\sum} \sigma^{2k}(}  \\ & & {\mathbf{PDV}_{g^{\prime}}(\mathbf{spec}_{{g^{\prime}}}({e_{1}}), \mathbf{spec}_{{g^{\prime}}}({e_{2}}))}  \\ & & {\ast_{\mathbf{choices}(e_{1}, e_{2})}}  \\ & & {\mathbf{PDV}_{g^{\prime\prime}}(\mathbf{spec}_{{g^{\prime\prime}}}({e_{1}}), \mathbf{spec}_{{g^{\prime\prime}}}({e_{2}})))}  \end{array} $$

#### The **PDV** Table

For a given gene vertex *g* and pair of species vertices *s*_1_,*s*_2_, the computation of **PDV**_*g*_(*s*_1_,*s*_2_) depends on the relationship between *s*_1_ and *s*_2_. There are three possible cases: (1) *s*_1_ and *s*_2_ are not ancestrally related, (2) *s*_1_=*s*_2_, and (3) *s*_1_ is ancestral to *s*_2_ (or vice versa). If *s*_1_ and *s*_2_ are not ancestrally related, then

$$\begin{array}{@{}rcl@{}} \mathbf{PDV}_{g}(s_{1}, s_{2}) & = & \mathbf{bothSDT}_{g}(s_{1}, s_{2}) \ +  \\ & & \underset{t_{1}\in\mathbf{losses}(s_{1})}{\sum} \sigma(\mathbf{PDV}_{g}(t_{1}, s_{2})) \ +  \\ & & \underset{t_{2}\in\mathbf{losses}(s_{2})}{\sum} \sigma(\mathbf{PDV}_{g}(s_{1}, t_{2})) \ -  \\ & & \underset{\substack{t_{1} \in \mathbf{losses}(s_{1}) \\ t_{2} \in \mathbf{losses}(s_{2})}}{\sum} \sigma^{2}(\mathbf{PDV}_{g}(t_{1}, t_{2}))  \end{array} $$

where **losses**(*g*,*s*) denotes the set of children *s*^′^ of *s* such that there exists a mapping node (*g*,*s*^′^) reachable from (*g*,*s*) through a single loss event. The first term is the pairwise distance vector for the case that both subtraversals begin with an SDT event. Otherwise, at least one subtraversal begins with a loss. The second term is the pairwise distance vector for the case that the subtraversal rooted at (*g*,*s*_1_) begins with a loss and the third is for the case that the subtraversal rooted at (*g*,*s*_2_) begins with a loss. Those vectors are shifted by one since one subtraversal incurs a loss that the other does not. The last term accounts for the overcounting that occurs when both (*g*,*s*_1_) and (*g*,*s*_2_) begin with losses.

If *s*_1_=*s*_2_ or *s*_1_ and *s*_2_ are ancestrally related (in which case we assume WLOG that *s*_1_ is ancestral to *s*_2_), a special case arises that requires a third DP table in order to avoid overcounting. This table, called **topSDT**_*g*_(*s*_1_,*s*_2_), computes the distance vector for the case that mapping node (*g*,*s*_1_) begins with an SDT event but (*g*,*s*_2_) begins with either an SDT or a loss event:

$$\begin{array}{@{}rcl@{}} \mathbf{topSDT}_{g}(s_{1}, s_{2}) & = & \mathbf{bothSDT}_{g}(s_{1}, s_{2}) +  \\ & & \underset{{t \in \mathbf{losses}(s_{2})}}{\sum}\!\!\!\!\!\!\! \sigma(\mathbf{topSDT}(s_{1}, t))  \end{array} $$

The first term accounts for pairs of subtraversals that both begin with SDT events, and the second term accounts for pairs where the subtraversal rooted at (*g*,*s*_2_) incurs a loss but the subtraversal rooted at (*g*,*s*_1_) begins with an SDT event. The shift in that term accounts for a contribution of one to the distance due to that loss event.

Now, if *s*_1_=*s*_2_, then let *s*=*s*_1_=*s*_2_. In this case:

$$\begin{array}{@{}rcl@{}} \mathbf{PDV}_{g}(s, s) & = & \mathbf{bothSDT}_{g}(s, s) +  \\ & & \underset{t \in \mathbf{losses}(s)}{\sum}\!\!\!\! \sigma(\mathbf{topSDT}(s, t)) +  \\ & & \underset{\substack{t_{1} \in \mathbf{losses}(s)\\ t_{2} \in \mathbf{losses}(s) \\ k = \delta(t_{1}, t_{2}))}}{\sum}\!\!\!\!\! \sigma^{2k}(\mathbf{PDV}_{g}(t_{1}, t_{2}))  \end{array} $$

The first term is the pairwise distance vector when both subtraversals begin with an SDT event. Otherwise, either one or both subtraversals begin with a loss. The second term accounts for pairs where one incurs a loss and the other begins with an SDT event. The third term is for pairs where both begin with a loss. If those losses are identical, they do not contribute to the distance and no shift is required. If those losses are distinct, they contribute two to the distance which results in shifting the distance vector by two.

Next, we consider the remaining case that *s*_1_ is ancestral to *s*_2_. This case is divided into two subcases. If *s*_2_ is not a child of *s*_1_ then:

$${}\mathbf{PDV}_{g}(s_{1}, s_{2}) = \mathbf{topSDT}_{g}(s_{1}, s_{2}) \ + \! \underset{t \in \mathbf{losses}(s_{1})}{\sum} \sigma(\mathbf{PDV}_{g}(t, s_{2})) $$

The first term accounts for the case that the subtraversal rooted at (*g*,*s*_1_) begins with an SDT event while the second term accounts for the case that (*g*,*s*_1_) begins with a loss; the shift in the summation accounts for a contribution of one to the distance due to that loss event.

The other subcase is that *s*_2_ is a child of *s*_1_. In this case, the subtraversal rooted at (*g*,*s*_1_) either begins with an SDT event, a loss event to mapping node (*g*,*s*_2_), or a loss event to a mapping node (*g*,*t*) where *t*≠*s*_2_. If it begins with a loss event *e* to mapping node (*g*,*s*_2_), the vector **PDV**_*g*_(*s*_2_,*s*_2_) undercounts the number of resulting pairs of subtraversals because a pair of subtraversals *A*,*B* that are both rooted at (*g*,*s*_2_) induce two pairs of subtraversals rooted at (*g*,*s*_1_) and (*g*,*s*_2_) respectively when *A*≠*B*: One pair is *e*,*A* with *B* and the other is *e*,*B* with *A*. Each pair *A*,*B* such that *A*≠*B* corresponds to an entry in **PDV**_*g*_(*s*_2_,*s*_2_) at index greater than 0. Therefore, we must increase those counts by a factor of two by introducing a vector operator *ρ*(*v*)=2*v*−*v*_0_. Now we have:

$$\begin{array}{@{}rcl@{}} \mathbf{PDV}_{g}(s_{1}, s_{2}) & = & \mathbf{topSDT}_{g}(s_{1}, s_{2}) +  \\ & & \sigma(\rho(\mathbf{PDV}_{g}(s_{2}, s_{2}))) +  \\ & & \underset{\substack{t \in \mathbf{losses}(s_{1})\\ t \neq s_{2}}}{\sum} \sigma(\mathbf{PDV}_{g}(t, s_{2}))  \end{array} $$

Finally, the recursively defined tables **PDV**, **topSDT**, and **bothSDT** are computed via dynamic programming by traversing the reconciliation graph in postorder (i.e., bottom up) so that when computing each table entry, the entries that are required for that computation have already been computed and saved.

### Asymptotic runtime analysis

Given a DTL-MPR instance (*S*=(*V*_*S*_,*E*_*S*_),*G*=(*V*_*G*_,*E*_*G*_),*ϕ*,*d*,*t*,*ℓ*), let *n*=|*V*_*S*_| and let *m*=|*V*_*G*_|. The reconciliation graph can be constructed in time *O*(*mn*^2^) [10]. In order to analyze the running time of computing the dynamic programming tables **PDV**, **topSDT**, and **bothSDT** used by the PDV Algorithm, we first show that the number of events in an MPR is bounded by *O*(*m*). From this result, it follows that the diameter is bounded by *O*(*m*) since, in the worst case, two MPRs could differ on every event.

#### **Lemma 1**

The number of events in an MPR is bounded by *O*(*m*).

#### *Proof*

First, note that a valid (although not necessarily minimum cost) reconciliation can be constructed by mapping *rg* to any leaf of *S* then, using only transfer events, the remainder of *G* can be embedded on the leaves of *S*, resulting in a reconciliation that incurs a cost of *t* for each of the internal vertices of *G*. Since *t* is some fixed positive constant, the total cost of the reconciliation is bounded by *mt*. This establishes a cost bound of *O*(*m*) on an MPR. Each internal vertex of *G* induces exactly one speciation, duplication, or transfer event and thus the total number of such events is *O*(*m*). Since an upper bound on the cost of an MPR is *tm*, and the loss cost *ℓ* is strictly positive, it follows that the total cost incurred by losses in an MPR is bounded by $\frac {mt}{\ell }$. Since *m* and *ℓ* are positive constants, the total number of losses is therefore bounded by *O*(*m*). Therefore, the total number of events in an MPR is bounded by *O*(*m*). □

It follows that the diameter of MPR space for the symmetric set distance metric is bounded by *O*(*m*) and thus all of the vectors maintained by the PDV Algorithm can be treated as having dimension *O*(*m*). Therefore, all vector addition, shift, and scale operations can be performed in *O*(*m*) time while convolution can be performed in time *O*(*m* log*m*) using the Discrete Fast Fourier Transform [18].

#### **Lemma 2**

For any pair (*g*,*s*), |**SDTevents**(*g*,*s*)|∈*O*(*n*).

#### *Proof*

There can be at most two speciation event nodes for (*g*,*s*) since *g*^′^ and *g*^′′^ can be associated with *s*^′^ and *s*^′′^ in two ways and there can be only one duplication event node. There can be *O*(*n*) transfer event nodes since one of *g*^′^ or *g*^′′^ must remain on *s* and the other can be transferred to at most one of *n* vertices of tree *S*. Thus, the mapping node (*g*,*s*) has a number of event node children bounded by *O*(*n*). □

#### **Lemma 3**

The PDV algorithm has worst-case running time bounded by *O*(*n*^4^*m*^2^ log*m*).

#### *Proof*

Computing **bothSDT**_*g*_(*s*_1_.*s*_2_) requires considering all pairs of SDT events in **SDTevents**(*g*,*s*_1_) and **SDTevents**(*g*,*s*_2_) and, for each combination, the dominant cost is due to the convolution. By Lemma 2, the number of pairs is bounded by *O*(*n*^2^) and, as a corollary of Lemma 1, the vector convolutions can be computed in time *O*(*m* log*m*). Thus, the total cost of computing **bothSDT**_*g*_(*s*_1_,*s*_2_) is bounded by *O*(*n*^2^*m* log*m*). Since there are *O*(*n*^2^*m*) entries in the **bothSDT** table, the total cost is bounded by *O*(*n*^4^*m*^2^ log*m*). It is easily verified that all of the other table entries require asymptotically less time, and thus the total running time of the algorithm is bounded by *O*(*n*^4^*m*^2^ log*m*). □

### Application to other distance metrics

The PDV algorithm was described for the symmetric distance metric but can be applied to other distance metrics as well. For example, in the *path distance* metric [12, 16, 17], the distance between two reconciliations *Φ*_1_ and *Φ*_2_ is defined to be $\sum _{g \in G} d_{S}(\Phi _{1}(g), \Phi _{2}(g))$ where *d*_*S*_(*s*_1_,*s*_2_) is the distance between vertices *s*_1_ and *s*_2_ in the species tree, *S*. The modification to the PDV algorithm for this metric simply replaces the 2*k* shift in the **bothSDT** computation by a shift equal to *d*_*S*_(*s*_1_,*s*_2_) to account for the distance incurred by mapping *g* to *s*_1_ in one MPR and mapping *g* to *s*_2_ in another. The maximum diameter under this distance metric is *O*(*mn*) and thus the vector operations now take time *O*(*mn* log*mn*). Therefore, the asymptotic running time for the PDV algorithm for this distance metric is *O*(*n*^5^*m*^2^ log*mn*). Similarly, the PDV algorithm can be easily adapted for other distance metrics such as the binary discrete distance metric [12].

## Experimental results

We implemented the PDV Algorithm in Python and validated the implementation by comparing the results to those found by a brute-force solver for all pairs of phylogenetic trees and leaf associations with up to six leaves and with a large sample of trees with up to 10 leaves. Our implementation, called *PairTree*, is available at www.cs.hmc.edu/ ∼hadas/pairtree.

We tested our code on a widely-used Tree of Life dataset comprising 100 primarily prokaryotic species and 4849 gene trees [5] using duplication, transfer, and loss costs of (2,3,1), (1,2,1), and (1,1,1) since these costs are frequently used in the literature [5, 19]. Although event costs (1,1,1) are perhaps the least likely to be biologically realistic, they induce very large reconciliation graphs which was useful for performance evaluation. Since the motivation for computing pairwise distance vectors is to understand large MPR spaces, we only considered gene families that induced at least 10^4^ MPRs.

### Runtime

We used a commodity server (AMD Opteron 6276 2.3 GHz, 503 GB RAM) for all of our experiments and running times are summarized in Table 1. Event costs (1,1,1) induce relatively large reconciliation graphs since all events have equal cost. In particular, an *n*^2^ factor in the *O*(*n*^4^*m*^2^ log*m*) worst-case analysis derived previously is due to an upper-bound of *O*(*n*) on the number of events associated with a given mapping node. In practice, most reconciliation graphs have relatively few events associated with each mapping node. However, there are cases where the number of event nodes can approach this bound for event costs (1,1,1) where all events have the same cost.

**Table 1:** Running time and average normalized distance with and without loss events

		Running time (seconds)			Normalized distance (w/ loss)		Normalized distance (no loss)	
DTL Costs	# Gene families w/ at least 10^4^ MPRs	Average	Standard deviation	Maximum	Average	Standard Deviation	Average	Standard Deviation
(2,3,1)	771	0.21	0.65	12.51	0.36	0.02	0.30	0.02
(1,2,1)	913	0.38	1.3	19.02	0.42	0.02	0.39	0.02
(1,1,1)	1492	3.53	14.25	295.90	0.42	0.03	0.41	0.03

For event costs (1,1,1), 3 of the 1492 gene families caused the algorithm to time out after five minutes and are not included in the statistics

### Distance statistics

We computed statistics on all pairwise distances across all gene trees that induced over 10^4^ MPRs. The *normalized distance* between a pair of MPRs is defined to be their distance divided by twice the number of internal vertices in the gene tree [11]. In the absence of loss events, the normalized distance is 0 if the two MPRs agree on all speciation, duplication, and transfer events and is 1.0 if the two MPRs disagree on all such events. Due to loss events, the normalized distance can exceed 1.0 in theory. Therefore, we also computed the normalized distances without losses so that the scale is from 0 to 1.0 and a distance of 1.0 implies disagreement on all speciation, duplication, and transfer events. Table 1 summarizes these results and demonstrates that, on average, MPRs disagree on a significant fraction of their constituent events, whether or not losses are considered.

### Pairwise distance distributions

**Fig. 2:**
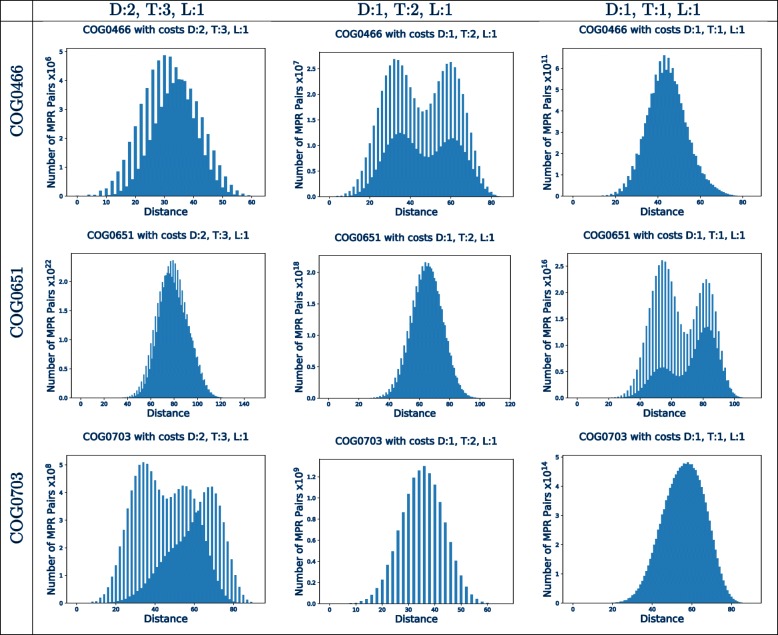
Pairwise distances (with losses) for three phylogenetic trees for three gene families in the Tree of Life dataset. COG0466 has 87 leaves, COG0651 has 84 leaves, and COG0703 has 85 leaves. All are reconciled to a species tree with 100 leaves. Each of the three rows corresponds to one gene family and the three columns correspond to the DTL cost parameters (2,3,1), (1,2,1), and (1,1,1), respectively. The entry at index 0 of each vector is omitted. These examples demonstrate that the pairwise distance distributions are sensitive to event costs and may be multimodal, indicating the presence of two or more clusters in MPR space

An important application of *PairTree* is in exploring the structure of the MPR space for a specific dataset of interest in order to determine the diversity of solutions and, ultimately, whether a single MPR is likely to be an adequate representative of the space. As an example, Fig. 2 shows the results from three gene trees that exhibit different distance distributions. These examples demonstrate that the pairwise distance distributions are dependent both on the trees themselves and on the event costs.

While many of the pairwise distance distributions are unimodal, there are also multimodal distributions which suggest the presence of distinct clusters of MPRs. The number of clusters cannot be directly ascertained from the number of modes in the distribution; MPR space is high-dimensional so a large number of clusters may induce only two modes.

Some distributions are comb-like with many pairs of MPRs at even distances and a smaller number of (or zero) pairs of MPRs at odd distance, or vice versa. This phenomenon is due to some MPRs having few or no losses. In the absence of loss events, the pairwise distance must be even by the definition of the symmetric set distance. Since losses result in shifting the distribution, comb-like distributions with odd distances are also possible.

## Conclusions

We have given an efficient polynomial-time algorithm for computing the pairwise distances between all maximum parsimony reconciliations in the DTL model that applies to several distance metrics. Further work is required to determine how these distributions should be interpreted with respect to the number of MPRs required to adequately represent the space. However, the mean and standard deviation alone, both reported by *PairTree*, provide important insights into the variation of solutions. Moreover, multimodal distributions are indicative of clusters of MPRs, which suggest that multiple MPRs are needed to adequately represent the space.

If there is evidence of clusters in MPR space, it is desirable to find a small set of representative MPRs, with at least one for each cluster. Ozdemir et al. [9] showed how Park’s *k*-medoid heuristic [20] and González’s *k*-centers 2-approximation algorithm can be adapted to find *k* medoids or centers, respectively. However, these algorithms have running times of the form *O*(*n*^*k*+3^ log*n*) rendering them viable only for small values of *k*. Thus, developing efficient clustering algorithms and methods for determining the appropriate number of clusters are important problems for future research.

## Data Availability

The software in this paper and the data used in the experiments are available at www.cs.hmc.edu/~hadas/pairtree.

## Abbreviations

DTL: Duplication-transfer-loss
MPR: Maximum parsimony reconciliation
PDV: Pairwise distance vector
